# Experienced time pressure and stress: electronic health records usability and information technology competence play a role

**DOI:** 10.1186/s12911-019-0891-z

**Published:** 2019-08-14

**Authors:** Tuulikki Vehko, Hannele Hyppönen, Sampsa Puttonen, Sari Kujala, Eeva Ketola, Johanna Tuukkanen, Anna-Mari Aalto, Tarja Heponiemi

**Affiliations:** 1 The Department of Information Services, National Institute for Health and Welfare (THL), P.O. Box 30, FI-00271 Helsinki, Finland; 2 Finnish Institute of Occupational Health, Helsinki, Finland; 3 Department of Computer Science, Aalto University, Espoo, Finland; 4 Emergency Unit, Central Finland Healthcare District, Jyväskylä, Finland

**Keywords:** Electronic health records, Information systems, Registered nurses, Time pressure, psychological distress, GHQ, general health questionnaire

## Abstract

**Background:**

Electronic health records (EHRs) are an elementary part of the work of registered nurses (RNs) in healthcare. RNs are the largest group of healthcare workers, and their experiences with EHRs and their informatics competence play a crucial role in a fluent workflow. The present study examined EHR usability factors and nurses’ informatics competence factors related to self-reported time pressure and psychological distress.

**Methods:**

A nationwide survey was conducted for working-age registered nurses in 2017. The study sample included 3607 nurses (5% men) in Finland. The association of age, sex, employment sector, EHR usability factors, and nurses’ informatics competence factors with time pressure and psychological distress were examined with analyses of covariance.

**Results:**

The EHR usability factors that were associated with high time pressure were low EHR reliability and poor user-friendliness. Regarding the nurses’ informatics competence factors, only low e-Care competence was associated with time pressure. Of the EHR usability factors, low EHR reliability and low support for cooperation were associated with high psychological distress. Of the nurses’ informatics competence factors, low e-Care competence was associated with high psychological distress.

**Conclusions:**

Unreliability and poor user-friendliness of EHRs seem to be prominent sources of time pressure and psychological distress among registered nurses. User-friendly EHR systems and digital tools in healthcare are needed. Nurses’ competence to use eHealth tools to tailor patient care should be strengthened through organizational and regional actions. For example, house rules about how to use eHealth tools and instructions on common practices in cooperation with other organizations could be useful.

## Background

Working life trends such as increased use of information and communication technology (ICT), constant learning of new skills, and time pressure place increasingly high demands on personnel [1]. Digitalization re-engineers organizational processes and routines, both encouraging and pressuring individuals to learn new skills and get accustomed to change [2]. Healthcare has not been isolated from the digitalization. In the field of healthcare, organizations, as well as individuals, need new skills and competence to cope with changing and increasingly digital work [3–6]. However, information technology competence or training to use new information technologies will not solve the dysfunction of information technology unless the technology itself functions well [7].

Research in the field of ICT functionalities and wellbeing in work in healthcare has focused on two main areas. First, several studies have focused on experiences and satisfaction with electronic health record (EHR) systems [8–10]. For example, in Texas and Norway, nurses’ satisfaction with their EHRs has been found to be low [3, 11, 12]. Among Canadian physicians, the ease of use of the patient information systems influenced their satisfaction and easy-to-use patient information systems were found to be more acceptable [13]. Among Canadian nurses, the most important determinant of their satisfaction was the compatibility of the EHR with the nurses’ preferred working style, existing practices, and values [14]. In Finland, physicians expressed more critical attitudes towards EHR systems when poor design, system slow-down and system downtime were common [15]. Second, many researchers have focused on work stressors in healthcare and studies indicate that nursing is a high-stress occupation and nurses are at risk of suffering from psychological distress [16, 17]. Time pressure among healthcare personnel has been recognized in many studies in Finland [18–21]. Time pressure has been found to decrease the quality of long-term care [16]. Quality deviations and insufficient time for completing required tasks (in other words, high time pressure) are related to elevated levels of stress of personnel. Recent studies suggest that time pressure and information systems related stress are crucial sources of stress also among Finnish physicians [20]. On the other hand, improved usability and technical stability of EHR systems lead to better work-related well-being, including lower time pressure [17].

Consequently, the interactive relationship between EHR usability factors and the well-being of nurses at work is interesting. For years information technology has been an elementary part of work in the healthcare, creating conductivity to the quality of healthcare [22]. Documentation using EHR systems comprises an integral part of the everyday work of nurses [3, 8, 23–25] and the use of information technology influences several elements of nursing care [9]. The advantages and problems related to specified EHRs have been described recently [10]. In some studies, nurses report that time needed for documentation has increased, although their ability to use templates for documentation reduced the time needed [26]. In the Finnish context, patterns of providing health services are changing. This change influences nurses’ work and the required competence in electronic recording within digital health services in many ways. Furthermore, the information technology that nurses use is under pressure for change. Currently, nurses have identified EHR systems’ poor stability and poor information system integration which leads to navigation between multiple screens as common problems [10]. In another study, navigating between multiple screens was identified as a usability barrier of EHR [27]. Difficulties in using the EHR system along with time pressure may increase psychological distress among nurses.

Furthermore, interplay between EHR usability factors and RNs’ informatics competence factors for both time pressure and psychological distress, is an area with little research and also national overviews of the issue are seldom available. Obviously, findings from observational studies with a wide range of EHR systems [3, 8–15] introduce variations regarding experiences related to EHR systems. In Finland, the largest professional group in health care is registered nurses and therefore it is interesting to find out their experiences of EHR systems. In this study, we do not limit on satisfaction with EHR systems, but consider the more important step, namely the RNs’ self-reported informatics competence.

### Aims of the study

The present survey study explores the associations of EHR usability factors and nurses’ informatics competence factors with self-reported time pressure and psychological distress among registered nurses.

## Methods

### The Finnish setting

In Finland, healthcare is funded mainly by taxation, and the municipalities are responsible for providing both primary care and secondary care services to their inhabitants [28]. Registered nurses (RNs) become licensed after 3–1/2 years of education at universities of applied sciences [28]. The expected skill needed in nursing includes digital and technical skills, language and cultural competence, and communication skills, especially in the context of a digital service environment [29]. Since 2000, the number of nurses has grown, and the number of nurses per 10,000 inhabitants is 14.6 in Finland. This is among the highest in the EU, while the EU average was 8.6 in 2014 [30]. Overall, registered nurses in Finland have more responsibilities for patient care compared to some other European countries [31]. This has been a component behind the findings that the Finnish healthcare system is fairly effective in international comparison. Nurses can work at different levels in the healthcare system. Secondary healthcare is provided by the hospital districts (21) owned by the municipalities (311 in 2017) and university hospitals in five areas make up the third level of the healthcare system specialization. The private sector includes mainly specialist clinics plus a few hospitals; but all intensive care units are in the public sector [28].

Coverage of EHRs is 100% in public healthcare, and the vast majority of private healthcare providers also use EHRs [32]. Standardized electronic nursing documentation was introduced in hospitals in order to increase patient safety and improve documentation [23, 25]. Within hospital districts, there are local systems for cooperation and information flow in addition to the national patient data repository (Kanta), which was launched between 2012 and 2017. The national e-prescription service was among the first of the national eServices. Currently, pharmacists, healthcare workers and the patient have access to the same information on prescriptions using Kanta, and healthcare workers and patients can access patient data stored in Kanta [33].

### The nationwide survey of EHRs use and RNs’ well-being at work

The nationwide survey conducted in 2017 aimed to study RNs’ experiences in EHR user and well-being at work in Finland. The survey instrument is available electronically in Finnish and Swedish [34]. The survey was targeted to all RNs who were members of the Finnish Nurses Association or The Union of Health and Social Care Professionals (TEHY) and were born in 1951 or later. An e-mail invitation to participate in a Web-based survey was sent via the two associations to 29,283 individual nurses. The invitation was followed by two reminder e-mails. A total of 3607 individuals responded—a response rate of 12%. The responses were representative of the population regionally as well as contextually (RNs working in hospitals, health centres, and private and social care) [10]. The survey related to RNs’ experiences of EHR use was based on respective physician surveys that gathered national-level information on physicians’ experiences three times since 2010 in Finland. The questions were adapted to nurses’ work where necessary, addressing various aspects of EHR use from the nurses’ viewpoint. A competence module was added for the RN survey, mapping the use of the Finnish Care Classification system (FinCC), the use of eHealth tools in tailoring patient care, electronic recording of patient data, and the ethical and safe way of using patient information systems. We have a special interest in nurses’ well-being at work, and questions on occupational well-being were added to the survey. The questions were selected from widely used measurements by a multidisciplinary group including RNs, researchers and psychologists. Ethical approval for the study was provided by the National Institute for Health and Welfare.

### Measurements

#### Time pressure and psychological distress at work

Time pressure was measured with two items from the Harris stress index [35, 36]: “Continuing time pressure and pressure from unfinished work” and “Too little time to do the job properly”. The items were framed by the question: “How often have you been distracted, worried, or stressed (during the past half-year period)?” The items are rated on a Likert scale ranging from 1 (never) to 5 (very often). The reliability for the sample was 0.91 (Chronbach’s α). Additionally, we measured psychological distress with four items (α = 0.86) from the General Health Questionnaire (GHQ) [37]. The response options ranged from 1 to 4. To avoid arbitrary cut-off points the scale was used as a continuous variable in this study. This was done also because we were more interested in general distress levels than the existence of minor psychiatric disorders [38, 39].

#### The EHR usability factors

A previous study identified a four-factor model of EHR-related usability factors [10]: EHR reliability, user-friendliness, impact on the quality of service, and support for cooperation. *EHR reliability* was measured using two items: “The system is stable in terms of its technical performance (no downtime)” and “The system quickly responds to commands”. Both items were rated on a Likert scale ranging from 1 (completely disagree) to 5 (completely agree) (α = 0.71).

*User-friendliness* was measured using nine items (α = 0.84). Two examples of the items are “Easy location of information” and “Nursing activities can be recorded smoothly”. The items were rated on a Likert scale ranging from 1 (completely disagree) to 5 (completely agree).

*Impact on quality of service* was measured using four items (α = 0.77). The items include “Information systems help improve the quality of care”, “Information systems help improve the continuity of care”, “Information systems help prevent medication errors”, and “Information systems help avoid duplicate examinations and laboratory tests”. Participants were asked to assess the benefits and disadvantages related to information systems. The items are rated on a Likert scale ranging from 1 (completely disagree) to 5 (completely agree).

*Support for cooperation* via information systems was measured using four items (α = 0.70) covering cooperation between nurses and doctors, between nurses in their organization, between nurses in different organizations, and between nurses and patients. Participants were asked how well they think the information systems support cooperation and communication between different parties. The items were rated on a Likert scale ranging from 1 (completely disagree) to 5 (completely agree).

#### Nurses’ informatics competence

The informatics competency question was: “How well do you feel you master the following skills required to use information systems?”, with 16 skills grouped into four factors. Firstly, the RNs’ competence related to the use of the Finnish Care Classification (FinCC) system was measured using four items (α = 0.95). The items were related to planning, implementation and evaluation of care needs, and the use of the care process according to FinCC. We call this factor *classification competence*. Secondly, the use of eHealth tools in tailoring patient care was measured using five items (α = 0.88), hereinafter *e-care competence*. Two examples of the items are “Supporting the customer choosing the services that best suit him” and “Working in a digital service environment”. Thirdly, the competence of electronic recording of patient data was measured using four items (α = 0.81), hereinafter *e-documentation competence*. Two examples of the items are “Patient classification system “and “Nursing summary”. Fourthly, competence in the ethical and safe way to use patient information systems was measured using three items (α = 0.75), hereinafter *e-ethics competence*. The items include, for example, “Use of data protection and information security principles in my daily work”. Participants were asked to assess their competence related to the use of information systems. All the competence items were rated on a Likert scale ranging from 1 (not at all) to 5 (very well).

#### Covariants

Age, sex and employment sector were included as covariates. The question of employment sector was obligatory; it was categorized as hospital, primary care, private practice, social care, and other.

### Statistical analysis

Analyses of covariance (ANCOVA) were conducted, with time pressure included as the dependent variable and age, sex, employment sector, EHR-usability factors (EHR reliability, user-friendliness, impact on quality of service, support for cooperation) and nurses’ informatics competence factors (classification competence, e-care competence, e-documentation competence, e-ethics competence) included as independent variables. The analyses were conducted in three steps. In the first step, age, sex and employment sector were used in the model (Model A). In the second step, factors related to patient information systems were added (Model B). Finally, factors related to nurses’ competence were added to the previous model (Model C). A similar series of ANCOVA was generated, in which psychological distress (GHQ) was included as a dependent variable. Because of incomplete data for some variables, the number of responses included in the statistical models varied. The analyses were conducted using IBM SPSS 24 statistical software.

## Results

The characteristics of the RNs by employment sector are shown in Table 1. Half (54%) of the participants worked in hospitals, and 24% worked in primary care. Approximately 13% of the respondents were RNs who had completed further education at a university of applied sciences or a university. Among the RNs, the non-standardized mean of time pressure was 3.79 and psychological distress, 2.02. The means of independent variables show that EHR reliability received the lowest scores, varying between 2.76 and 3.17 by employment sector. Nurses’ e-ethics competence got the highest scores (mean ranging between 4.36 and 4.57).

**Table 1:** Characters of the study sample according to employment sector. Electronic health record (EHR) usability factors include reliability, user-friendliness, impact on quality of service and support for cooperation. Registered nurses informatics competence factors enclose classification (of Finnish Care Classification system), and electronic care, documentation and ethics

Employment sector							
	Total n (%)	Hospital n (%)	Primary care n (%)	Private n (%)	Social care n (%)	Other n (%)	p*
Education of registered nurses							
Intermediate school	1200 (34.9)	647 (35.0)	284 (34.7)	78 (39.6)	170 (35.1)	21 (23.6)	< 0.001
University of applied sciences	1806 (52.5)	934 (50.6)	470 (57.4)	90 (45.7)	265 (54.6)	47 (52.8)	
Additional education at a university of applied sciences or a university	431 (12.5)	266 (14.4)	65 (7.9)	29 (14.7)	50 (10.3)	21 (23.6)	
Sex							
Women	3233 (94.9)	1724 (94.1)	784 (97.0)	188 (94.5)	461 (96.0)	76 (86.4)	< 0.001
Men	174 (5.1)	108 (5.9)	24 (3.0)	11 (5.5)	19 (4.0)	12 (13.6)	
Mean (SD)							
Age	46.2 (10.99)	45.7 (11.23)	47.0 (10.71)	47.5 (9.97)	46.6 (10.71)	45.8 (11.93)	
Time pressure^a^	3.79 (0.99)	3.80 (0.98)	3.87 (0.98)	3.55 (0.96)	3.81 (0.98)	3.29 (1.17)	
Psychological distress ^b^	2.02 (0.71)	2.00 (0.71)	2.00 (0.73)	2.07 (0.75)	2.09 (0.72)	1.92 (0.75)	
*EHR usability in terms of* ^a^							
Reliability	2.85 (0.92)	2.76 (0.90)	2.80 (0.91)	3.14 (0.90)	3.17 (0.93)	2.92 (0.95)	
User-friendliness	3.03 (0.71)	2.97 (0.69)	3.05 (0.70)	3.15 (0.76)	3.20 (0.73)	2.93 (0.76)	
Impact on quality of service	3.20 (0.78)	3.18 (0.77)	3.16 (0.79)	3.27 (0.82)	3.34 (0.75)	3.31 (0.85)	
Support for cooperation	3.06 (0.75)	3.04 (0.72)	3.10 (0.76)	3.01 (0.99)	3.07 (0.80)	3.19 (0.73)	
*Competence related to* ^a^							
Classification	3.94 (0.96)	3.93 (0.97)	3.96 (0.92)	3.76 (0.99)	3.99 (0.96)	3.66 (1.24)	
e-Care	3.61 (0.89)	3.52 (0.89)	3.68 (0.86)	3.67 (0.90)	3.76 (0.90)	4.00 (0.70)	
e-Documentation	4.19 (0.70)	4.21 (0.68)	4.15 (0.71)	4.17 (0.73)	4.22 (0.72)	4.09 (0.77)	
e-Ethics	4.39 (0.64)	4.38 (0.65)	4.39 (0.61)	4.36 (0.65)	4.42 (0.64)	4.57 (0.53)	

^a^Range 1–5; ^b^Range 1–4

Table 2 shows the results of the covariance analyses for time pressure and psychological distress. The EHR usability factors that were associated with high time pressure were low EHR reliability and low user-friendliness. Moreover, low e-care competence was associated with time pressure. Working in primary healthcare was also associated with high time pressure.

**Table 2:** The results of the covariance analyses for time pressure and psychological distress

	Model A^a^			Model B^b^			Model C^c^		
	F	p	R^2^	F	p	R^2^	F	p	R^2^
Time pressure			0.008						
Age	0.03	0.867		1.324	0.250		0.855	0.975	
Sex	1.62	0.251		2.347	0.126		2.816	3.213	
Employment sector	5.68	< 0.001		5.949	< 0.001		5.990	< 0.001	
*EHR usability in terms of*						0.057			
Reliability				24.844	< 0.001		23.749	< 0.001	
User-friendliness				14.244	< 0.001		13.384	< 0.001	
Impact on quality of service				1.634	0.201		0.097	0.756	
Support for cooperation				1.856	0.173		1.262	0.261	
*Competence related to*									0.083
Classification use							0.004	0.950	
e-Care							17.585	< 0.001	
e-Documentation							3.607	0.058	
e-Ethics							0.007	0.932	
Psychological distress			0.001						
Age	3.64	0.057		6.751	0.009		6.715	0.010	
Sex	0.03	0.854		0.070	0.791		0.002	0.968	
Employment sector	1.24	0.290		2.079	0.081		1.082	0.364	
*EHR usability in terms of*						0.033			
Reliability				5.263	0.022		5.641	0.018	
User-friendliness				4.765	0.029		2.236	0.135	
Impact on quality of service				1.924	0.166		0.501	0.479	
Support for cooperation				12.092	0.001		9.456	0.002	
*Competence related to*									0.056
Classification use							0.334	0.563	
e-Care							12.547	< 0.001	
e-Documentation							0.020	0.887	
e-Ethics							0.625	0.429	

^a^Model A included age, sex, and employment sector.

^b^Model B included age, sex, employment sector, EHR reliability, user-friendliness, impact on quality of service, and support for cooperation.

^c^Model C included age, sex, employment sector, EHR reliability, user-friendliness, impact on quality of service, support for cooperation, classification competence, e-care competence, e-documentation competence, and e-ethics competence

Age, low EHR reliability and low support for cooperation were associated with high psychological distress. In addition, low e-care competence was associated with high psychological distress (Table 2).

## Discussion

For this study, it was of interest to investigate both EHRs usability factors and nurses’ informatics competence factors associations to self-reported time pressure and psychological distress. The results of the present study revealed that low reliability and poor user-friendliness of EHRs were associated with time pressure among RNs in Finland. Moreover, low reliability and low support for cooperation and information flow were associated with high levels of psychological distress. Additionally, low competence in using eHealth tools in tailoring patient care and working in primary care were associated with high time pressure as well as high levels of psychological distress. The age of RNs was associated with high levels of psychological distress but not with time pressure. Similar results suggesting improving competency in the use of information and communication technologies in the delivery of patient care has been reported among practicing nurses in Canada [40], the Netherlands [7] and Finland [6]. Furthermore, training needs are widely discussed as part of age-related management [41].

Rapid technological progress puts nurses on a continuous learning track. Digital competence comprises not only IT skills but also the ability to meet complex demands using psychosocial resources (including skills and attitudes) in a particular circumstance [5]. For patients with multiple care needs, nurses should tailor care between social care and healthcare [42, 43]. In these situations, a fluent exchange of information between professionals and organizations is important for care continuity. It is estimated that one in 10 healthcare patients needs help for a range of problems, but most working-age people need services only occasionally [44]. Working-age people are also more likely to master the use of digital services [45]. In everyday work, healthcare professionals need training and coaching in adopting new digital services for patient work [6]. It is important to note that teaching patients to use digital services is a new task for healthcare professionals.

Our results related to high time pressure among RNs’ work echoes the general trend of increasing time pressure in working life [46]. Specifically in RNs’ work, the technical problems of EHRs were associated with higher time pressure, as was the situation among Finnish physicians [17]. To summarize, both poorly functioning electronic health records and information technology competence play a role in high time pressure and high levels of psychological distress among RNs. Therefore, strategies for improving both challenges are needed. Firstly, healthcare professionals’ experiences with EHR systems should be taken into account in the development of these systems. Secondly, the organizations should invest in informatics education for RNs. Involving professionals in the design of EHR systems or other information technology development seems to take place mainly in discussions [3, 4, 47]. This is an unfortunate state of affairs since user experiences should be a key component of information technology system development. Based on job-demands theory [48], the lack of opportunities to influence working practices likely decreases well-being at work [47, 49].

The present study was not without limitations. First, reaching the informants via an e-mail survey proved difficult and the response rate remained low. This was the first Finnish survey related to RNs’ experiences in EHR user and well-being at work and in the next survey in 2020, the informing of coming survey needs to intensify. However, the study presents a cross-sectional study of a large sample of RNs in Finland. Second, we used self-reporting measures: Related to nurses’ wellbeing at work, these were well-known and validated; and related to EHR-related factors, measures were consistent with factors from the cross-sectional studies of physicians. Nevertheless all of the EHR-related factors also showed good reliability and in the analysis, we controlled many variables, such as age, sex and employment sector, but of course, some other variables may impact to time pressure and psychological distress too. Third, thus our findings give a picture of RNs working in Finnish healthcare the results of the research are not directly generalizable; but the results should be tested in other healthcare systems using different kinds of EHR systems or varying ways of organizing healthcare in the various implementation stages of EHR systems.

The EHR systems, the users, the work organization and other social and healthcare providers involved in patient care interact with each other. Simple technical solutions for challenges in healthcare are no longer sought, and the expectations regarding EHR systems as the single element that solves the problems in the management of patient care have turned from optimistic to pessimistic [50]. Comparing with the widely used survey instrument [51] to that we used in this study both instruments measure mostly the same elements, but additionally we measured the reliability of information systems (e.g. system downtime), which was associated to high time pressure. To fix the usability problems of EHR systems, the first important step is to identify them [3, 10, 47]. For example, on the individual level, navigating between multiple screens has been identified as a usability barrier, and poor stability as a potential source of patient safety problems [3, 17, 27, 52, 53]. Likewise, in tailoring the training to the personnel, it is crucial to first ask and identify their opinion on what important skills they are missing [7]. It must also be remembered that information technology competence is not merely about an individual’s skills, but also about the organization-level habits of working processes and practices that are shared with other healthcare professionals [6]. The management of new working processes requires a clear vision and goal communication, management support, effective information on the implementation of the service and its implementation benefits, as well as the involvement of professionals [54].

Building care pathways for typical patient cases is an option to strengthen integrated care for patients in modern digital healthcare. This would also ease the nurses’ decision-making process in care management. In a sustainable solution to improving EHR usability should involve healthcare organizations, including front-line staff, working with the EHR software developers. Descriptions of care pathways may provide a shared platform and understanding to integrate the patient treatment points and flow of information between the patient and professionals within an organisation and if needed between organisations [47]. In addition, political toolkits like the Nursing Association’s e-Health Strategy try to emphasize RNs’ eHealth competence and participation in development work. Healthcare providers and politicians should care about improving the working life of those who deliver care in order that they achieve better care, better health at the population level, and lower healthcare costs [55]. All in all, user-friendly EHR systems and digital tools are needed, along with training and coaching personnel in using digital services in tailoring patient care to help improve patient-centred care.

## Conclusions

The main achievements, including contributions to the field, can be summarised as follows: In the Finnish context, this is the first national-level survey focused on RNs’ experiences of EHRs. Moreover, few studies have focussed on EHRs usability factors and nurses’ informatics competence. Unreliability and poor user-friendliness of EHRs seem to be prominent sources of time pressure and psychological distress for RNs. The largest group of healthcare workers in Finland is RN’s and organisations should ensure that their experiences with EHRs and digital tools become visible for the EHR software developers. RNs’ competence to use eHealth tools to tailor patient care should be strengthened through organizational and regional actions.

## Data Availability

The data are available in aggregate level through database report system (only in Finnish) (https://sampo.thl.fi/pivot/beta/fi/steps2/sh/fact_steps2_sh) and the data used from the corresponding author on reasonable request.

## Abbreviations

EHR: Electronic health record
FinCC: Finnish Care Classification system
GHQ: General Health Questionnaire
RN: Registered Nurse
